# Role dimensions of practice nurses and interest in introducing advanced nurse practitioners in general practice in Ireland

**DOI:** 10.1002/hsr2.555

**Published:** 2022-03-09

**Authors:** Mary Casey, Laserina O'Connor, Daniela Rohde, Liam Twomey, Walter Cullen, Áine Carroll

**Affiliations:** ^1^ UCD School of Nursing, Midwifery and Health Systems Dublin Ireland; ^2^ UCD School of Medicine and Medical Science College of Health and Agricultural Sciences Dublin Ireland; ^3^ UCD School of Medicine Dublin Ireland

**Keywords:** general practice, nurse, public health, primary care

## Abstract

**Background:**

Internationally many countries have implemented strategies to enhance primary care, to strengthen their health systems to cope with an aging population, the rise of chronic conditions, and increased costs. Primary care has the potential to address these challenges, however, general practitioners are increasingly struggling to meet patient demand resulting from a growing and aging population. Expanding the role of general practice nurses to advanced nurse practitioner (ANP) level has worked internationally and could equally be a solution to the Irish context. However, their current role must first be established as well as their level of interest in becoming an ANP.

**Aim:**

To explore the role of general practice nurses and their interest in becoming an ANP.

**Design:**

A survey design.

**Method:**

A purposeful sample of general practice nurses (*n* = 40) was undertaken between April and June 2019. Data were analyzed using the Statistical Package for Social Science (SPSS V 25.0; IBM).

**Results:**

General practice nurses appear to have an agenda in relation to activities associated with wound care, immunizations, respiratory and cardiovascular issues. Just over half of the respondents were not interested in becoming an ANP. Their perceived challenges associated with the implementation of the role include a lack of support from general practitioners, a lack of resources, insurance issues, and a lack of understanding of the role. Challenges were associated with undertaking further training and their experience of having more work transferred to general practice without concomitant reallocation of resources.

**Conclusion:**

General practice nurses have extensive clinical experience to deliver major improvements in primary care. Educational opportunities need to be provided for upskilling existing general practice nurses to advanced practice level. Greater understanding of the role and the potential contribution of the role in general practice is required among medical colleagues and the public.

## 1 INTRODUCTION

In 2018, the Astana declaration^1^ reaffirmed the global commitment to strengthening primary healthcare as it was seen as inclusive, effective, and efficient way to enhance people's physical, mental health, and social well‐being. Primary care teams, commonly led by general practitioners (GPs), provide comprehensive family and community‐oriented medical care. Primary care systems worldwide are being challenged by an aging population, an increasing number of chronic diseases, and economic constraints while expected to respond to increasing demands for services.^2^ Fundamental to the provision of high‐quality primary care is the general practice nurse who typically provides a wide range of services. This higher demand for health care services necessitates a cultural shift from specialist hospital‐centric care models to community‐led person‐centered integrated care and, in many countries, advanced nurse practitioners (ANPs) are emerging as a potential solution for these challenges.^3^

### 1.1 Background

The essential feature of general practice nursing is employment within a general practice setting working alongside a GP. In Ireland, the Sláintecare policy^4^ supports the reorientation of healthcare out of the hospital setting and into the community delivered at the lowest level of complexity, appropriate to the individuals' needs. Sláintecare policy has highlighted the burden on service demands associated with chronic disease and its management has been described as suboptimal, fragmented, and siloed. Around 3005 GPs provide care to Ireland's population of 5‐m people.^5^ It is suggested by Homeniuk and Collins^6^ that GPs undertake approximately 25 consultations daily. Yet only half of the country's GP practices are operating to full capacity and have identified that they cannot take on new patients because of working part‐time, staff shortages, and lack of holiday cover. In Ireland, GPs are self‐employed under a variety of individual contracts with the Health Service Executive for the provision of services to either exclusively public patients, or private patients or to a public–private mix.^7^

Although there is mounting evidence of the positive impact of nurses on patient outcomes,^8^ there is resistance to enhancing the role. In Ireland, the general practice nurse, is employed privately by the GP and the current salary subsidy is provided by the tax payer via the Primary Care Reimbursement Service of between €31–38K and the GP pays the indemnity cover. A comparison of the educational requirements, years of experience, and indicators of competency are provided in Table 1. The difference in the competence exercised by each individual is demonstrated in the implementation of the scope of practice.

**Table 1:** The educational requirements and indicators in the six domains of competence for a general practice nurse and an advanced practitioner in Ireland

Registered general nurse	Advanced nurse practitioner^9^
BSc. Nursing (4‐year degree program) (NFQ level 8)	BSc. Nursing (4‐year degree program)
No specified accredited postregistration education	Master's Level qualification (or higher) (NFQ level 9)
No minimal postregistration clinical experience requirement	Minimum of 2 years postregistration clinical experience
Formal qualifications as a nurse responsible for general care shall provide evidence that the professional is able to apply: “(a) competence to independently diagnose the nursing care required using current theoretical and clinical knowledge and to plan, organize and implement nursing care when treating patients; (b) competence to work together effectively with other actors in the health sector, including participation in the practical training of health; (c) competence to assist individuals, families, and groups towards healthy lifestyles and self‐care; (d) competence to independently initiate life‐preserving immediate measures and to carry out measures in crises and disaster situations; (e) competence to independently give advice to, instruct and support persons needing care and their attachment figures; (f) competence to independently assure the quality of, and to evaluate, nursing care; (g) competence to comprehensively communicate professionally and to cooperate with members of other professions in the health sector; (h) competence to analyze the care quality to improve his own professional practice as a nurse responsible for general care.”^10^ p. 14 All competencies encompass six domains of practice	The competencies for the ANP build on the competencies achieved/acquired to register as a nurse with the Nursing and Midwifery Board of Ireland in each of the 6 practice domains. A specific standard is set for each domain to:
	1. Apply ethically sound solutions to complex issues related to individuals and populations 2. Utilize advanced knowledge, skills, and abilities to engage in senior clinical decision making 3. Actively contribute to the professional body of knowledge related to his/her area of advanced practice 4. Negotiate and advocate with other health professionals to ensure the beliefs, rights, and wishes of the person are respected 5. Manage risk to those who access the service through collaborative risk assessments and promotion of a safe environment 6. Lead in multidisciplinary team planning for transition across the continuum of care
1. Professional values and conduct competencies 2. Clinical decision‐making competency knowledge and cognitive competencies 3. Communication and interpersonal competencies 4. Management and team competencies 5. Leadership and professional scholarship competencies	

A practical example illustrating the role of a staff nurse and an ANP in rheumatology is highlighted in Table 2.

**Table 2:** The role of a staff nurse and an advanced nurse practitioner in rheumatology^9^ (p. 29)

Domain of practice	General practice nurse	Advanced nurse practitioner
Knowledge and cognitive competencies	Develops knowledge of the pathology and diagnosis of rheumatology‐related illnesses. Ability to communicate information to clients and their family regarding the current stage of illness	Teaches nursing and medical staff about new theories. Develops awareness of new evidence‐based treatments within nursing and interdisciplinary team. Discuss with the client relevant investigations and treatment options that are acknowledged by their peers as exemplary. Provide clinical leadership by demonstrating advanced theoretical knowledge and clinical skills in managing defined rheumatology conditions
Sample of extended skills/registered nurses (ONMSD October 2016) Electrocardiograph (ECG); male catheterization; suprapubic catheter insertion; noninvasive ventilation; swallow assessment; nurse prescribing (medicinal products); percutaneous endoscopic gastrostomy reinsertion; venesection	Sample of the list of tasks and activities provided by the practice nurse^11^ ECG Health promotion such as dietary advice Chronic disease management Women's health such as cervical screening Nurse prescribing Management duties such as managing clinic activities Audit and research Counselling 24 h blood pressure monitoring	Extract,^9^ the advanced nurse practitioner [in general practice] works as part of the team supporting G.P.s across the country to manage patients with a stable/chronic disease in the community. They also guide the less well‐controlled patients through the health system providing them with the resources to self‐manage their illness and if necessary refer for expert care. The advanced practitioner provides timely access to expert care and information and support to patients in prevention activities which includes meeting monitoring and prescription needs. The model of care is a shift from hospital‐based care to care in the community and reduces the length of hospital stay

Table 2 shows that the advanced practitioner level moves beyond developing knowledge of the pathology to teaching interprofessional colleagues, from communicating information to patients to discussing treatment options thus moving from a competency to a capability model.

### 1.2 The role of ANPs in primary care

Reviews on the effectiveness of nurse‐led clinics in primary care found that such models of care delivery improved patient satisfaction, reduced hospital admission, and mortality rates.^12^ A recent review concluded that nurse‐led care for common minor health conditions was as effective and less costly than GP Care.^13^ A Cochrane review suggests that task shifting from doctors to nurses is a useful strategy to improve access, efficiency, and quality of primary care.^14^ Primary care services led by nurses in advanced practice have demonstrated to be as safe and effective as those led by physicians^15^ and providing improved access for people with minor health problems.^16^ A recent economic evaluation of ANPs versus GP's in treating common conditions found that direct costs of nurse practitioner consultations were significantly less than those of GP consultations.^17^

Yet, according to van der Biezen et al.,^18^ improvement in quality of care and the ability to offer technical care were important driving factors for GPs to employ ANPs. Moreover, the bespoke employee arrangements give rise to a lack of standardization of the activities of general practice nurses, and this is compounded by the absence of a national standardized preparation for this role. In addition, the way in which general practice is organisationally structured and the hierarchical position of the general practice nurse limits opportunities for knowledge sharing.^19^ These are important considerations in the context of implementing professional practice standards for practice nursing.^20^ There is also a lack of promotion and continuing professional development opportunities for practice nursing and general practice nurses can experience a strong sense of professional isolation in comparison to nurses employed in the public sector. Indeed, While and Webley‐Brown^21^ describe nurses in general practice feeling undervalued and dissatisfied with working conditions. When this is added to negative power relations resulting for their status as employees, gender issues and the part‐time nature of the role, the unique challenges of general practice nurses' role and the importance of adequate role preparation become clear. The rising health care costs, and physician shortages increase demand for a review of the services provided by the GPs and particular consideration for the employment of ANPs.^3^ In Ireland, a recent reform of GP and service development agreement was concluded^22^, ^23^ with emphasis on chronic disease management. While there is mention of practice nurses there is no mention of ANPs.

Consideration is currently being given to the development of advanced clinical practitioners which, according to Ljungbeck and Sjogren Foss^24^ allows more time for the doctors to see people. In Ireland, this proposed new role in primary care is known as a community paramedic who will work “collaboratively with other partners in health to bring the right care to the right patient in a home setting.”^25^ (p. 2) The idea is these practitioners will work autonomously under the guidance of the GP. It would seem that an opportunity is wasted and effectiveness and efficiency are to be ignored by developing a new role in primary care rather than employing more general practice nurses and upskilling them to ANP, which is known to have a positive impact on patient outcomes.

For many medical consultants, both the role and the scope of practice of the ANP are unclear.^26^ This is also true in relation to understanding the role and scope of practice of general practice nurses, where little is known about the ways in which they are deployed within each practice.^27^ Indeed, many GPs and general practice nurses themselves do not have a clear understanding of the role or the scope of practice with few distinguishing tasks from roles.^28^, ^29^ Therefore, the complexity of the general practice nursing role can remain hidden, and they can be an unsung hero of the team.^21^ In addition, Jakimowicz et al.,^30^ found that experienced GPs do not think nurses are autonomous and many general practice nurses articulated their role as an assistant to the GP rather than as an independent professional responsible for their own clinical practice. Moreover, some GPs can mistakenly assume that they are responsible for everything that transpires within the practice which can give the impression that general practice nurses are relieved of any accountability arising from their own decisions.^30^ Securing the future of general practice nursing requires a willingness to do things differently building on examples of approaches already undertaken to develop ANP services in acute care. However, before this can take place the current role of general practice nurses must first be established. The aim of this study was to address this gap.

## 2 METHODOLOGY

A survey method was used.

### 2.1 Sample

A purposeful sample of 40 GPNs from the membership of the Irish Practice Nurses Association was undertaken with prior permission obtained from the members to ensure adherence to General Data Protection Regulations. A mixture of urban and rural postal addresses from one national hospital group were selected. Inclusion criteria specified that the general practice nurse must be in current employment.

### 2.2 Data collection

Data from the postal survey were collected between April and June 2019. The survey questionnaire together with a letter of invitation, an information leaflet and a stamped addressed envelope were distributed via the postal service to each of the 40 general practice nurses.

### 2.3 Survey instrument

The survey contained 40 nursing activities using the preprepared list of practice nursing activities guided by previous research.^11^, ^31^ These activities related to dealing with patients and families, chronic disease management, prescribing, providing advice, and other activities. Demographic details were also collected. Respondents were only required to indicate a “yes” or “no” if they undertake the activity. Respondents were also asked about the types of continuous professional development (CPD) courses they undertook and two open questions were included on how they perceived the role of the ANP in terms of its contribution and potential implementation challenges in general practice. Interest in becoming an ANP was rated on a scale of 1 (*not at all interested*) to 10 (*very interested*). To enhance the face validity of the survey, a small pilot study to test the understanding of the survey was conducted with two general practice nurses, two GPs, and one ANP. No changes were made to the wording of the survey which took about 15 min to complete.

### 2.4 Ethical considerations

Permission for the study was received from the university (Approval no. LS‐19‐28‐XXX). Return of the completed questionnaire was accepted as informed consent.

### 2.5 Data analysis

Data were analyzed using the Statistical Package for Social Science (SPSS V 25.0; IBM). Descriptive statistics, including means (SD) and percentages were used to summarize and interpret the data. The qualitative open questions were analyzed for common themes by the lead researcher who is a registered general nurse.

## 3 RESULTS

### 3.1 Survey sample characteristics

The response rate was 48% (*n* = 19) with 42% (*n* = 8) currently employed at a staff nurse grade, 42% (*n* = 8) employed either at clinical nurse manager grade 1 or 2. The most common qualifications were a higher diploma (31.6%, *n* = 6) and bachelor's degree (31.6%, *n* = 6). Approximately 37% (*n* = 7) were dually qualified either as a registered nurse and midwife or as a registered general nurse and a children's nurse. Respondents had been working in their current general practice for a mean of 7.9 years (SD = 5.8, range: 0.4–17), with a mean of 24.9 years (SD: 10.7) of experience since first registering as a nurse. Demographic details and registration status of participants are presented in Table 3.

**Table 3:** Demographics and registration status of general practice nurses respondents

Demographics		*N* (%)
Female		19 (100%)
Age mean (SD, range)		50.6 (9.3, range: 36–70)
Nationality		
Irish		16 (84.2%)
Another European Union country		3 (15.8%)
Education		
Diploma in nursing		5 (26.3%)
Bachelor's degree		6 (31.6%)
Hospital certificate (specialist training)		2 (10.5%)
Higher diploma in nursing		6 (31.6%)
General practice location (*n* = 18)		
Urban location		10 (55.6%)
Rural location		4 (22.2%)
Mixed location		4 (22.2%)
Registration status		
Length of time working in current general practice		Range: 0.4–17 years, *M* = 7.9 SD = 5.8
Number of years of nursing or midwifery experience since first registration		Range: 19–47 years, *M* = 24.9, SD = 10.7
Registered general nurse only		53% (*n* = 10)
Registered general nurse and registered midwife		21% (*n* = 4)
Registered general nurse and registered children's nurse		16% (*n* = 3)
Registered general nurse and registered nurse prescriber		5% (*n* = 1)
Registered general nurse and clinical nurse specialist		5% (*n* = 1)

### 3.2 Dimensions of the role

General practice nurses appear to have an agenda in relation to chronic disease management in relation to wound care, respiratory and cardiovascular issues such as raised blood pressure which were at the top end of selected activities as seen from Table 4.

**Table 4:** List of role activities undertaken by practice nurses in order of frequency

Role activities	Example	Yes	No
		*N* (%)	
Providing patient information leaflets		19 (100.0)	
Dressings/wound care/suture removal		19 (100.0)	
Providing telephone advice		18 (94.7)	1 (5.3)
Immunizations		18 (94.7)	1 (5.3)
Cervical smears		18 (94.7)	1 (5.3)
Management of Automated External Defibrillator/emergency bag		18 (94.7)	1 (5.3)
Diabetes review		17 (89.5)	2 (10.5)
Ear wax removal		17 (89.5)	2 (10.5)
Phlebotomy		17 (89.5)	2 (10.5)
Ambulatory blood pressure monitor fitting		17 (89.5)	2 (10.5)
Nebulisation		16 (84.2)	3 (15.8)
12 lead electrocardiograph		16 (84.2)	3 (15.8)
General data protection regulation compliance		16 (84.2)	3 (15.8)
Evaluate existing practice		16 (84.2)	3 (15.8)
Develop proposals for change		16 (84.2)	3 (15.8)
Providing investigation results		15 (78.9)	4 (21.1)
Liaison with public health nurse		15 (78.9)	4 (21.1)
Implementing evidence‐based care		15 (78.9)	4 (21.1)
Identify, report, manage critical incidents		15 (78.9)	4 (21.1)
Demonstrate leadership skills		15 (78.9)	4 (21.1)
Counseling		14 (73.7)	5 (26.3)
Use of national chronic illness guidelines		14 (73.7)	5 (26.3)
Repeat depot drugs/monitoring (contraception)		13 (68.4)	6 (31.6)
Networking with local practices		12 (63.2)	7 (36.8)
Participate in continuous professional development		12 (66.7)	6 (33.3)
Ability to refer to hospital		11 (57.9)	8 (42.1)
Liaison with state agencies		9 (47.4)	10 (52.6)
HeartWatch		9 (47.4)	10 (52.6)
Disease coding		9 (50.0)	9 (50.0)
Perform audits		9 (47.4)	10 (52.6)
Liaison with primary care team members		8 (42.1)	11 (57.9)
Ante/postnatal care		7 (36.8)	12 (63.2)
Spirometry		6 (31.6)	13 (68.4)
Repeat depot drugs/monitoring (psychiatry)		6 (33.3)	12 (66.7)
Attend primary care team meetings		6 (31.6)	13 (68.4)
Participate in research		5 (27.8)	13 (72.2)
Running warfarin clinic		4 (21.1)	15 (78.9)
Organizing home help, allowances		3 (16.7)	15 (83.3)
Prescribing initial agreed items, for example, nonsteroidal anti‐inflammatory drugs		2 (10.5)	17 (89.5)
Prescribing agreed repeat items, for example, oral contraceptive pill		2 (10.5)	17 (89.5)

*Note*: Respondents were asked to identify other activities not listed in the questionnaire. These included the following:

Administrative—patients for apts. Injections and so forth. Sexual health screening. Asthma reviews—adult and child. Blood pressure review. Wound review and dressing. Chaperone to patients with GP. Vaccines. Vaccines ordering. Protocol and policy writing. Clinical meetings with team. Cervical smear taking, cryotherapy, audiometry, counseling, mindfulness techniques, dietary advice. Group education for diabetes, Venesection for hemochromatosis. Print repeat prescriptions for GP to sign. Urinalysis, stocktaking, and ordering, Triage emergency walk‐in patients before patient being seen by GP.

Approximately 84% (*n* = 16) engage in evaluation of practice and provide proposals for change and see themselves as implementing evidenced‐based care (78.9%, *n* = 15). Less than fifty percent (47.4%, *n* = 9) engage in audit and even less (27.8%, *n* = 5) engage in research. Two respondents (10.5%) appeared to be engaged in prescribing activities, yet only one respondent is a registered nurse prescriber. Noteworthy is that two‐thirds of the sample (66.7%, *n* = 12) stated that they engaged in CPD activities. Only 6 respondents provide an explanation of the types of CPD activities and these CPD activities included “lots of one day courses, course on diabetes, cardiovascular, smear testing, ear irrigation, introduction to practice nursing courses, asthma and chest pain clinic.”

### 3.3 Interest in becoming ANPs

Table 5 indicates respondents' interest in becoming an ANP in general practice.

**Table 5:** Interest in becoming an advanced nurse practitioner in general practice

	Frequency	%	Cumlative percent
Not at all interested	5	26.3	26.3
2	1	5.3	31.6
3	2	10.5	42.1
4	2	10.5	52.6
6	1	5.3	57.9
7	3	15.8	73.7
9	1	5.3	78.9
Very interested	4	21.1	100.0
Total	19	100.0	

**Figure 1:**
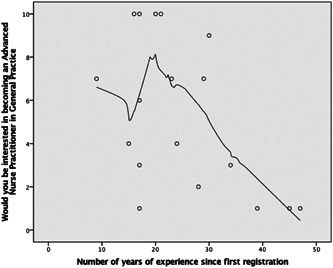
The relationship between years of experience and level of interest in becoming an ANP in general practice. ANP, advanced nurse practitioners

All respondents (*n* = 19) answered this question. The mean score was 5.1 (SD = 3.6, range: 1–10 with just over half of participants (52.6%, *n* = 10) giving a response of between 1 and 4, indicating that they were not interested in becoming an ANP in general practice. As seen in Figure 1, general practice nurses with more years of experience appear to be less likely to be interested in becoming ANPs: *r* = −0.527, *r* ^2^ = 0.278, *p* = 0.025. Figure 1 indicates the level of interest scale is from 1 to 10 on the left side and suggests there may be a short peaking of interest after 15 years postregistration experience, it seems that this interest declines thereafter. However, caution must be exercised due to the sample size.

### 3.4 Contribution and challenges that an ANP role would bring to general practice

When asked about the perceived contribution that an ANP would bring to the practice the following comments were proffered by nine respondents. One said it “would be very beneficial. Reduce workload for Drs. Enhance pt care, quick and accurate service. Empowering for nurse.” Another stated the ANP role would give “more autonomy to the nurse leading to more effective and efficient care of patients. For example, transfer to hospital or other services would free up the medical staff time to concentrate on other tasks.” Another respondent stated it would “time saving and [lead to] better time management” while a fourth respondent stated that the ANP role would enable the practice to “run independent clinics with support of prescribing doctor.” A fifth respondent claimed the role would lead to “providing the highest standard of care and evidence‐based care for the patients and staff, the practice, and assisting and supporting the GPs in a role that currently isn't being filled.” Both the sixth and seventh respondents stated that the ANP role would lead to “better care” and “reduce waiting times to see the GP and more job satisfaction [and] greater competency.” According to respondent eight the ANP role would “be valuable for running clinics such as diabetes, asthma, hypertension” and the ninth and final respondent stated the following “I feel an ANP would only work if the practice has more than 2 GPs and 2–3 nurses….”

In relation to the challenges an ANP might encounter in general practice, 11 respondents provided the following written comments in answer to this open question as highlighted in Table 6.

**Table 6:** Perceived challenges that an ANP might encounter in general practice

Challenges you think an advanced nurse practitioner in general practice might encounter
Time constraints as it is we are already booked to the hilt on a daily basis with all health promotion, chronic illness prevention etc. Diabetes cycle of care. I personally think that we have enough to do with education and prevention without prescribing also. A practice nurse diploma, degree, etc. etc. would be so much more beneficial
I think the practice would be very open to a nurse practitioner. The more the nurse can do the better!
Resistance to change—some GPs may take issue [with] that nurse and this new role and jobs. Resources may be lacking to assist nurse. Lack of understanding of nursing staff capabilities. Services being referred to not recognising nurse's role in care of patients
Autonomy
Expectations by some patients to be seen by the doctor. Perhaps some prejudice from some GPs
Reluctance of GP to allow expansion of ANP. Restriction and support in GP practice to expand the role of ANP
No support from GPs. Insurance issues. No recognition from HSE—resistance from patients
Who does the job the practice nurse did previously?? Too many roles for the advance nurse practitioner not enough paid time off work to complete training
What is the difference then between a GP and an advanced nurse practitioner in general practice? Would the lines be blurred in their actual job descriptions?
The ongoing deliberate transfer of hospital work into under‐resourced general practice
overload of work—unless you have a team of nurses

Abbreviations: ANP, advanced nurse practitioner; GP, general practitioner.

Challenges that an ANP might encounter range from time constraints to resistance and lack of support from the GP to a lack of resources, to insurance issues, a lack of understanding of the role, managing patient expectations. Respondents also mentioned challenges associated with undertaking further training and the experience of having more work transferred to general practice without concomitant reallocation of resources.

## 4 DISCUSSION

### 4.1 Role dimensions of practice nursing

Results show that general practice nurses have extensive clinical experience and provide a valuable health care resource in general practice. They perceive their role as centered on activities related to immunizations, chronic disease management, undertaking reviews of patients with diabetes, respiratory cardiovascular issues such as hypertension. This relates positively with Harrington et al.^31^ who found that Irish GPNs had more extended roles than their UK counterparts, specifically in chronic disease management and family planning. Indeed, Halcomb et al.,^32^ found that most of the activities that general practice nurses engaged with were in‐keeping with the role of an ANP. Mobilization of general practice nursing workforce and movement to ANP roles can strengthen general practice, while providing benefits and safe care for patients.^14^, ^33^, ^34^, ^35^ Immunizations and health screening featured frequently on the survey results which really speaks to current public knowledge that general practice nurses are integral to the continuation of services and the delivery of the COVID‐19 vaccination program.^36^ Respondents also indicated a high level of engagement in activities such as health promotion vis a vis providing information leaflets and offering telephone advice. Similar tasks of practice nurses were identified by Matthys et al.,^37^ consistent with patient education, and technical nursing skills.

### 4.2 Introducing ANP to general practice

Just under half of the sample were interested in becoming an ANP and, while based on a small sample size it appears, respondents with more years of experience were less interested in becoming an ANP. This may be reflective of the age group and experience level of the respondents and this finding also relates to previous research^38^ who found that early‐career nurses had the greatest interest in becoming ANP. This finding may also be related to the challenges to implementing the role which were associated with, resistance to change, insurance concerns, a lack of resources, a lack of adequate preparation and ongoing educational support, and a lack of understanding of their scope of practice. Jakimowicz et al.,^30^ also reported concerns about ambiguity about the scope of practice of ANPs as a challenge to role enhancement. Going forward, encouraging general practice nurses to gain specialist or advanced knowledge, attitude, and skills and using them at work can motivate them to stay in general practice.^39^ Evolution of the role of nurses in general practice to a more autonomous decision‐making role can be facilitated by a clear vision, mission, team communication, and trust‐based interprofessional relationships^40^ within a bespoke educational development plan. Therefore, these nurses need to be adequately prepared and assisted to upskill to advanced practitioner levels. Fundamentally, having a vision and understanding of the role of each professional in primary healthcare would be invaluable to create opportunities for knowledge sharing.^41^ Such knowledge sharing would also assist in reducing resistance to change due to poor understanding of the ANP role. In addition, general practice nurses do not routinely hold their own professional indemnity and hence it has been difficult to progress the role until practical and policy issues such as insurance and indemnity cover are addressed. Similar concerns were highlighted by Bury et al.,^42^ who suggest that a lack of funding for training and for posts and indemnity concerns accounts for the highest potential barriers to an enhancement of the role of practice nurses. Financial considerations were also previously identified.^28^, ^43^, ^44^, ^45^

While general nursing graduates are often unaware of the different cultures of primary care in comparison to hospitals^46^ because undergraduate nursing education offers no clinical experience in practice nursing, respondents in this study were well able to highlight the potential contribution of the ANP role. This included a more empowered, autonomous nursing role, enhanced patient care, a positive impact on the workload of the GP, and improved patient outcomes. However, despite the evidence supporting the contribution of ANPs to patient care, particularly in acute hospital‐based care,^47^, ^48^, ^49^, ^50^, ^51^, ^52^, ^53^ and the positive impacts of ANPs on patient outcomes,^8^ the potential to advance the role of general practice nurses remains unexplored. In the main, the identified challenges to implementation of the ANP role included the lack of adequate resources and a lack of shared understanding of the role. This lack of understanding of the ANP role has been previously identified.^28^, ^30^, ^54^ Moreover, it can act as a barrier to implementation of more expanded roles^55^ and, in the context of the current study, these findings may contribute to an explanation of the semi‐strong interest in becoming an ANP in general practice. GP recruitment is unlikely to provide enough GPs to meet the ever‐increasing demand in the near future, so it is imperative that more general practice nurses are employed and that they have the option of up‐skilling to address the workload.^39^

### 4.3 Study limitations

Some bias which may have arisen due to a small sample size and the study being conducted in just one geographical health area. Therefore, the robustness of the findings must be tempered with these limitations. Moreover, the survey instrument consisted of a list of tasks without any indication of the actual nursing roles associated with the task itself. The task list reveals what the nurses do but not their opinion and confidence to work in an advanced role or any issues with power imbalance in a GP setting. Reliability of the survey was not undertaken, and this may have resulted in the nursing aspect of the role largely remaining hidden in favor of more easily observed tasks taken from the literature. Our study is also prone to self‐reporting bias and recruitment bias since respondents reported on their own activities and achievements and respondents were selected from the membership of one professional nursing organization. Nevertheless, this study gives an understanding of the current dimensions of the role of general practice nurse and provides, a view of their current interest in becoming an ANP in general practice in Ireland and their perception of the challenges and contributions of such a role.

### 4.4 Implications for clinical practice

Enabling general practice nurses to work to their full scope of practice will help mitigate future workforce shortages and improve patient access to care. Introducing the role of ANP to general practice can be realized by providing bespoke educational opportunities for existing practice nurses to upskill or by attracting suitably prepared ANPs to work in general practice.

### 4.5 Implications for future research

Research is required to further explore the barriers to implementation of the ANP role in general practice beyond what is indicated in this study and how GPs can support the implementation of APNs in primary care settings. It would also be worthwhile to examine the level of confidence of general practice nurses to work in an advanced practice role.

## 5 CONCLUSION

Data from this study has shown that general practice nurses engage in nursing activities appropriate to chronic disease management which may be at the ANP level. Furthermore, they have insight into the potential contribution of ANPs and anticipate resistance to the role from GPs. With greater knowledge and understanding of the scope of practice and the role of ANP, resistance to the implementation of the role could be managed and their contribution to patient care manifest. Therefore general practice nurses and GPs, should be enabled to fully capitalize on the contributions of ANPs to provide a mechanism for the provision of integrated care and to ensure increased access to healthcare resulting in overall health service improvement. Establishing a primary care‐specific career pathway for ANPs in general practice is essential as they are an essential component of the primary healthcare team and are necessary for the development of the specialty.

## CONFLICT OF INTERESTS

The authors declare no conflict of interest.

## ETHICS STATEMENT

Approved by the Ethics Committee of the Human Research Ethics Committee—from University College Dublin.

## AUTHOR CONTRIBUTIONS

*Conceptualization*: Mary Casey, Laserina O'Connor, Liam Twomey. *Data curation, validation*: Mary Casey, Daniela Rohde. *Formal analysis, investigation, project administration, supervision*: Mary Casey, Daniela Rohde, Laserina O' Connor. *Methodology*: Mary Casey, Laserina O' Connor, Liam Twomey, Daniela Rohde. *Writing–original draft preparation*: Mary Casey, Aine Caroll, Walter Cullen. *Writing–review and editing*: Mary Casey, Daniela Rohde, Aine Caroll, Walter Cullen. All authors have read and approved the final version of the manuscript. Mary Casey has full access to all the data in this study and takes complete responsibility for the integrity of the data and the accuracy of the data analysis.
